# Integrating specimen databases and revisionary systematics

**DOI:** 10.3897/zookeys.209.3288

**Published:** 2012-07-20

**Authors:** Randall T. Schuh

**Affiliations:** 1 Division of Invertebrate Zoology, American Museum of Natural History, New York, New York 10024 USA

**Keywords:** specimen databases, workflows, revisionary systematics

## Abstract

Arguments are presented for the merit of integrating specimen databases into the practice of revisionary systematics. Work flows, data connections, data outputs, and data standardization are enumerated as critical aspects of such integration. Background information is provided on the use of “barcodes” as unique specimen identifiers and on methods for efficient data capture. Examples are provided on how to achieve efficient workflows and data standardization, as well as data outputs and data integration.

## Introduction

Meier and Dikow (2004) argued that biodiversity data should come from revisionary studies–rather than from uncritical digitizing of museum specimen data, because such revisions 1) provide the most accurate identifications, 2) provide the most complete taxonomic coverage, 3) and they satisfy these points in a cost-effective way. Nonetheless, revisions are what might be viewed as the traditional approach to creating a database of specimens for a taxon. In the following pages I will provide a rationale and a roadmap for satisfying both the acquisition of high-value biodiversity data while at the same time creating a structured database of that same information during the revisionary process.

The creation of specimen databases–a subset of a field that has frequently been referred to as biodiversity informatics (Johnson 2007)–has reached a point in its maturity that has brought down per-specimen digitization costs and increased accessibility of available tools to a much broader range of systematists than was the case 15 years ago. Movement into the Internet Age, the more widespread use of digital technologies such as barcodes, and the increasing sophistication and availability of database technology are all contributing factors.

One manifestation of the maturity of biodiversity informatics can be seen in the United States National Science Foundation (NSF) program Advancing Digitization of Biological Collections (ADBC 2011), a ten-year initiative designed to promote and fund the digitization of biological collections. The core digitization activities are in Thematic Collection Networks (TCN), funded projects that bring together a group of collections focusing on a common research or investigative theme. The TCNs are coordinated through a “national resource” or HUB (Home Uniting Biocollections). Through the activities of the HUB we should anticipate seeing the dissemination of more tools and improved access to relevant technology and the methods by which data can be integrated across collections and which would also be of use to revisionary systematists.

Most of the tools applied in specimen data capture—such as databases and barcodes–were initially developed for use in industry. Their application in the realm of biological collections was originally in collection management, rather than as an adjunct to the preparation of scientific publications such as taxonomic revisions. Even though the technology is available, the full integration of biodiversity databases into revisionary studies is far from a fully realized objective. The reasons may include the foreign nature of the technology to older investigators, the lack of direct access to the tools, the lack of technical expertise for implementation of the technology, and simple reluctance to alter traditional approaches to the preparation of revisions.

In the following pages I will argue for the adoption of database tools as an integral part of the revisionary process. This is not just an argument for the adoption of modern technology. Experience suggests that the benefits accrued will more than justify the costs incurred, both in terms of money spent to acquire the necessary equipment and software as well as time spent learning to incorporate “databasing” into one’s day-to-day taxonomic labors.

I have already written about aspects of this subject in two prior papers which focused on the methods for the solution of large-scale taxonomic problems (Cassis et al. 2007) and the use of Web-based data capture as a model for multi-national systematic research projects (Schuh et al. 2010). The lessons learned, and approaches outlined, in those papers derived largely from experience gained in the conduct of an NSF-funded Planetary Biodiversity Inventory (PBI) project (http://research.amnh.org/pbi/ ) for the study of the plant-bug subfamilies Orthotylinae and Phylinae (Insecta: Heteroptera: Miridae). As was the case in those works, this paper is based largely on approaches developed during the PBI project. The present paper will not attempt to resolve the intertwined issues of 1) whether databases should be collection based, with research data gathered from across a spectrum of such information repositories, 2) whether databases should be project based and integrate data across taxonomic lines or research themes, or 3) whether both types of databases can and should co-exist. Rather, I will focus on workflow, data connections, data outputs, and data standardization, issues that are central to enhancing the revisionary taxonomic process.

## Database choice

The arguments to be made in this paper assume that one has access to a specimen database with certain “basic” features. These include the capability to efficiently capture all relevant and necessary data in a highly structured format, the capability to organize those data in ways useful to the reviser, and the capacity to output data for direct use in revisions as well as for the production of maps and other visual aids. A number of such database products exist, some free of charge, and most capable of performing the necessary functions. They exist as stand-alone products, as institutional tools functioning on a local area network, or as Internet-based tools. Because information on these databases is not the primary intent of this article, and because the logic of choice is beyond the scope of this article, I will not dwell further on the issue database choice. As sources of further information the reader might wish to consult Schuh et al. (2010) and the abstracts in Session 1 from the 2011 meeting of the Entomological Collections Network (http://www.ecnweb.org/dev/AnnualMeeting/Program ).

## Unique specimen identifiers (USIs)

**Figure 1.**
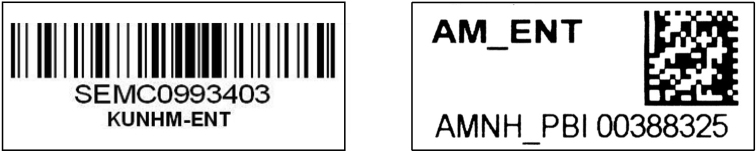
Linear barcode label (left), matrix code label (right).

The use of *barcodes* to uniquely identify individual specimens goes back at least to the work of Daniel Janzen and the InBio collections in Costa Rica (Janzen 1992). In the intervening 20 years, code technology has advanced, such that many applications now use matrix codes (Fig. 1, right) which can store much more information in a smaller format than is the case with linear barcodes (Fig. 1, left). Whatever technology you choose, the use of unique specimen identifiers (USIs) provides the capacity to track individual specimens with exactitude, and to directly associate a variety of information sources with them.

Machine readability, although not an essential component of a USI, is a valuable aspect of barcode and matrix code labels. At $250 or less, the cost of code readers is now about one-tenth what it was in 1994 (Thompson 1994), making them a truly affordable databasing asset. The most convincing argument for the use of machine reading is that the readers do not make mistakes, whereas human transcription is prone to error. Once their use becomes part of your work routine, barcode readers significantly enhance the speed and accuracy with which USI data can be entered into the database, either when doing original data entry or when retrieving specimen data. Some have worried that barcode reading technology will change over time, and that encoded labels will therefore become obsolete. In anticipation of this potential reality, all such labels should include the alphanumeric representation of the code as well as the code itself (Fig. 1).

Production of barcode labels can be contracted out to specialized suppliers or can be done in house. Because of the widespread use of the technology, appropriate tools for their preparation and printing are readily available. Nonetheless, a distinct difference between the commercial application of these technologies and their use in biological collections is that the latter group of users expects the labels to be permanent, suitable for alcohol and dry storage, and for the printed matter to be of high resolution, whereas none of those criteria is important in industrial applications such as package delivery and airline baggage identification. Although most any printer can be used to print barcodes, specialized software is required to produce individual labels with sequential numbering (e.g., BarTender 2012). Many database applications expect coded information to be in a certain format. Thus, when preparing barcodes, it is important to verify that the format of the code, such as the institutional acronym/collection code and numerical string that follows, are in a format accepted by your database.

Curators of biological collections have long applied catalog numbers to specimens, although such practice has been much less common with insect collections than with those of recent vertebrates, fossils, and plants, for example. Although these “catalog” numbers were often not unique within institutions, let alone across institutions, they did offer a way to uniquely associate specimens with log-books of data, accession information, field notebooks, and other written resources. Most barcode implementations come much closer to globally-unique identification than was the case with traditional catalog numbers, through the use of codes that combine an institution code + a collection code + plus a catalog number. This approach complies the with Darwin Core standard promoted by the Taxonomic Database Working Group (2012), with the caveat that a single code is sometimes applied to a group of specimens, often referred to as a *lot*, in which case the unique identifier applies to more than one specimen.

The use of barcodes has resulted in the frequent attachment of multiple codes to individual specimens, often in addition to traditional catalog numbers. Several factors are at play, including the use of barcodes as the modern equivalent of catalog numbers as well as to identify specimens used in independent research projects. Sometimes these two uses are included in a single label, sometimes on separate labels. Recent Internet-based discussions suggest that prevailing opinion regards the attachment of multiple labels as acceptable, often unavoidable, and that the all of the codes should remain on the specimens in perpetuity. Some or all of these codes may be globally unique.

## Verbatim vs Transformed Data: A choice mediated by the use of USIs

A recent symposium organized for the 2011 meeting of the Entomological Collections Network (Reno, Nevada; http://www.ecnweb.org/dev/AnnualMeeting/Program ), included a more or less equal number of presentations arguing for 1) the verbatim capture of all label data in a single text field with subsequent transformation into a more highly structured format, or for 2) transformation of label data into a publication-ready format as an integral part of the data-capture process. Schuh et al. (2010) made the argument for the latter approach, but to my knowledge there are few 1) published arguments concerning the merits and demerits of these alternative approaches or 2) quantitative studies analyzing the efficiency of the alternative approaches.

Verbatim data capture allows for data acquisition with minimum training of the data-entry personnel. The only real requirement would seem to be the ability to read the labels and convert them into a text string. Those data must then be transformed into a structured format and written to the database tables by the use of some software algorithm or other automated data-parsing approach. Finally, the accuracy of the transcription must to be checked, an additional step, and one that will require greater expertise in interpretation of label data than did the initial data entry.

Transforming data as part of the data-capture process, so that the data are in the exact form used by the database requires additional training of personnel over what is needed for verbatim data capture. Nonetheless, because the data are structured during the process of data capture, these data are ready for straightforward review for accuracy, at which point they can be considered “publication ready” and the additional training effort will be available for all subsequent data capture.

Even though errors may be made under either approach, the use of USIs allows for subsequent investigators to return to individual specimens with substantial confidence concerning the correspondence of original and transcribed data. It is my view, and that of many of my colleagues, that the capture of transformed data is more efficient because it is a one step process that allows for immediate use of the data. Data captured en masse from collections will not be available until they have undergone algorithmic transformation and been approved for upload, thus potentially presenting a time lag that will hinder the progress of the reviser or other data user.

## Data-capture Work Flow in Revisionary Studies

### Label generation: Capture field data to the database and generate all labels from it

Many specimens used in revisionary studies, possibly most particularly in entomology, come from the dedicated fieldwork of the reviser. Thus, the opportunity to use appropriate technology in conjunction with fieldwork would seem to be a straightforward choice. This would include the capture of latitude/longitude and altitude data in the field through the use of a GPS (global positioning system) device in the form used by geographical information systems software and the recording of field data in exactly the format to be used in the specimen database. Thus, the choice should be degrees and decimal parts thereof for lat/long data and meters for altitude. Locality and collection-event data can be directly captured in digital form in the field, or recorded to an archival field notebook and captured in digital form at the earliest subsequent opportunity. GPS data can be downloaded directly, an approach that precludes mistakes during transcription of numbers, one of the most common errors made in the capture of field data.

The argument for using a database to capture/store field data and to produce specimen labels is bolstered by the many examples of specimens in collections where multiple collectors on the same field trip produced their own labels. Although such labels contain similar information, they are frequently not identical and thus may end up in a database as representing distinct localities. The drawbacks are one or more of the following: 1) what was actually a single locality will likely end up being georeferenced multiple times, or if lat/long data were captured in the field, those data may still not be identical on the labels; 2) one or more renderings of the collection locality may contain errors; 3) the locality may be easily interpreted in one rendering but difficult to interpret in another; and 4) some of the labels may be substandard from a curatorial point of view. Using the database from the outset, including for the generation of labels, facilitates data standardization and the uniform presentation of data in all of its subsequent uses. It also greatly facilitates the retrospective capture of data for specimens whose localities are already in the database. This last point has economic implications, because even though the personnel time available to enter all specimens collected at a given locality may not be available at the time the specimens are mounted and labeled, the cost of entering just the locality/collection event data at the time of the fieldwork will never be an issue.

### Specimen data: Enter specimen data early in the revisionary process

Although it has been said many times, and therefore may seem trite, the use of a database can save many key strokes. Once the data have been entered and checked for accuracy for a given locality, they can be re-used in the generation of labels, for preparation of reports of “specimens examined”, and for many other purposes. If for any reason an error is found, it can be corrected and all subsequent and varied uses of those data will be accurate and uniform. The capture early on in the revisionary process of as much specimen data as possible allows for the structuring and examination of those data in ways that are otherwise difficult and cumbersome. What is paramount is that the data are captured once but useable in many ways without the need for re-keyboarding. Nonetheless, it is probably fair to say that in the traditional preparation of a revision, the last thing to be done was to capture specimen data, whether using a word-processing file, spreadsheet, or relational database. The use of a specimen database facilitates the capture of specimen data much closer to the beginning of the revisionary process, so that all relevant observations on specimens can be managed through the medium of the database and available over the entire course of the revisionary process. In addition to locality data, such observations might include host data, habitat descriptions, museum depository information, dissections, images, measurement data, and DNA sequence files, to name just some of the possibilities.

### Capturing specimen data: Organize specimens before capturing data

**Figure 2.**
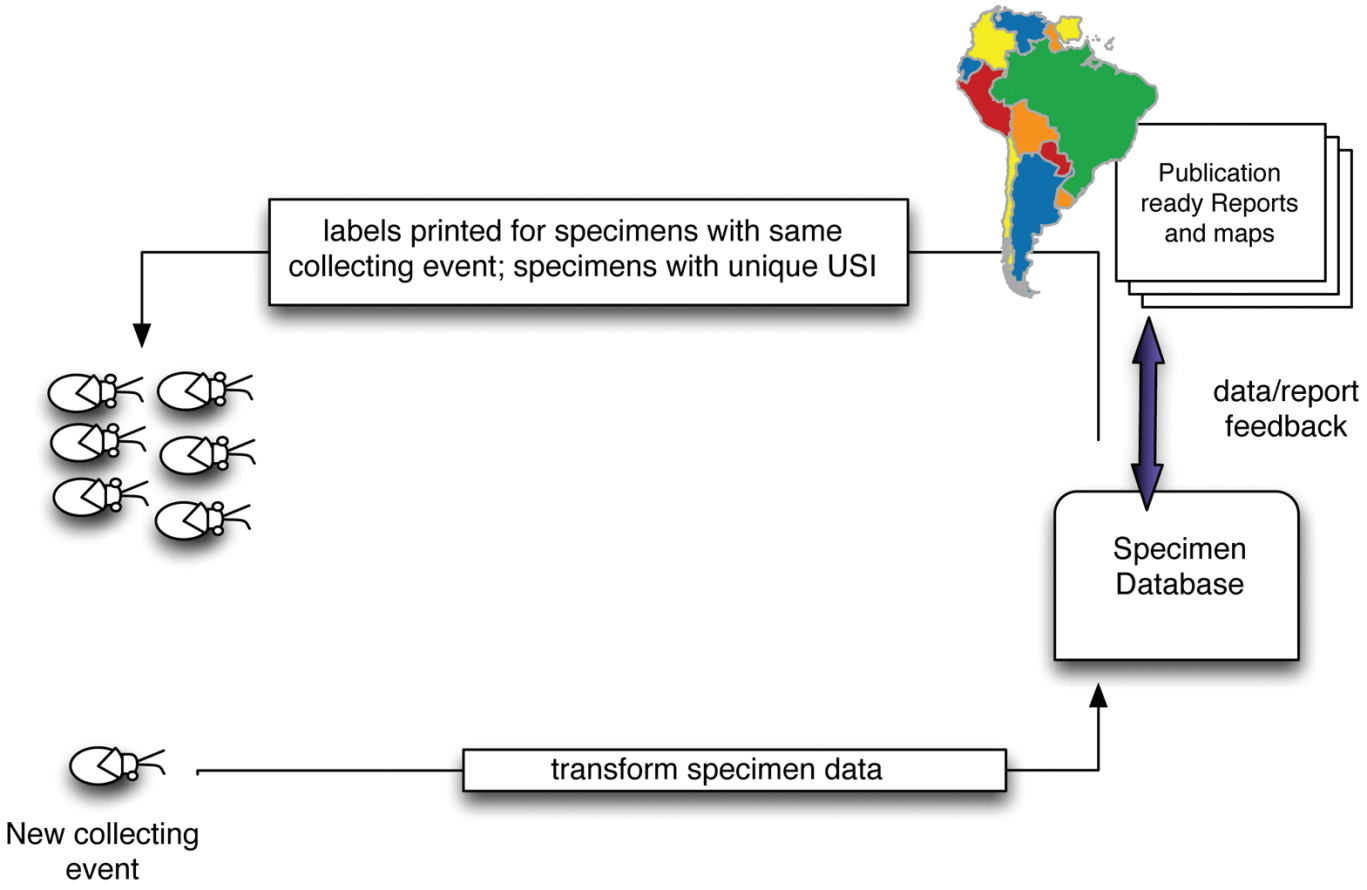
Diagram of specimen data connections and work flows.

With some forethought and advance preparation the process of retrospective specimen data capture can be made more efficient and also facilitate other aspects of the revisionary process. Collective experience of participants on the Planetary Biodiversity Inventory project, and other colleagues, recommends the following sequence of events for dealing with specimens from any given institution (Fig. 2):

1. Sort specimens by provisional species criteria (morpho species, etc.)

2. Sort specimens by locality

3. Sort specimens by sex

4. Affix sequential unique specimen identifiers (barcodes, matrix codes)

5. Enter data in database

This workflow is efficient because it allows for series of specimens of the same species, sex, and locality to bear USI codes in sequential order and for data for all of those specimens to be captured as a single action. Of course, this approach is most important in those cases where there are multiple examples of a species from a single collecting event.

Although sexing specimens may not be necessary or possible for all taxa, in many groups the standard description is based on one sex, or the other. Sorting by sex before specimen data are entered facilitates comparisons, adds a logical aspect to the organization of the material in collections, and helps to produce sequential USIs, which saves space in presenting data on specimens examined. If during the course of preparing a revision specimens are found to have been initially misidentified, the records for those specimens can be readily retrieved via the barcode and the identifications in the database can be corrected.

## Data Connections

### Georeferencing and mapping: Using the database as an analytic tool

Georeferencing–the addition of latitude/longitude data to individual specimen records–permits the mapping of specimen distributions in space. Such mapping should be part of the revisionary process, rather than taking place near the end, as has traditionally been the case. As a matter of standard practice, lat/long data should be available on all specimen labels being produced as a result of fieldwork in this day and time. And, as mentioned above, data from modern fieldwork should desirably be captured to a database for the preparation of all labels, such that no manual georeferencing will be required. Under this approach, georeferencing is intimately related to the issue of workflow, because the earlier in the revisionary process the specimen data can be mapped, the more useful they will be. Nonetheless, lat/long data will have to be determined for legacy material.

Georeferencing was at one time a time-consuming and tedious process. It is now much easier, due to the ready availability of automated tools such as GeoLocate (2010), unrestricted access to quality gazetteers for much of the world (Fuzzy Gazetteer 2003, Geonames: http://www.geonames.org/ , GNIS 2011), and the universal accessibility of Google Earth (2012) and Google Maps (2012), among other sources. Thus, there is a strong argument for georeferencing of specimen data in close coordination with initial capture of those data. Such an approach will allow for the visualization of distributions early in the revisionary process. This will provide a feedback loop concerning the accuracy of the georeferencing itself, the interpretation of distributional patterns, and the on-the-spot investigation of suspect identifications as recognized by the visualization of distributional outliers.

**Figure 3.**
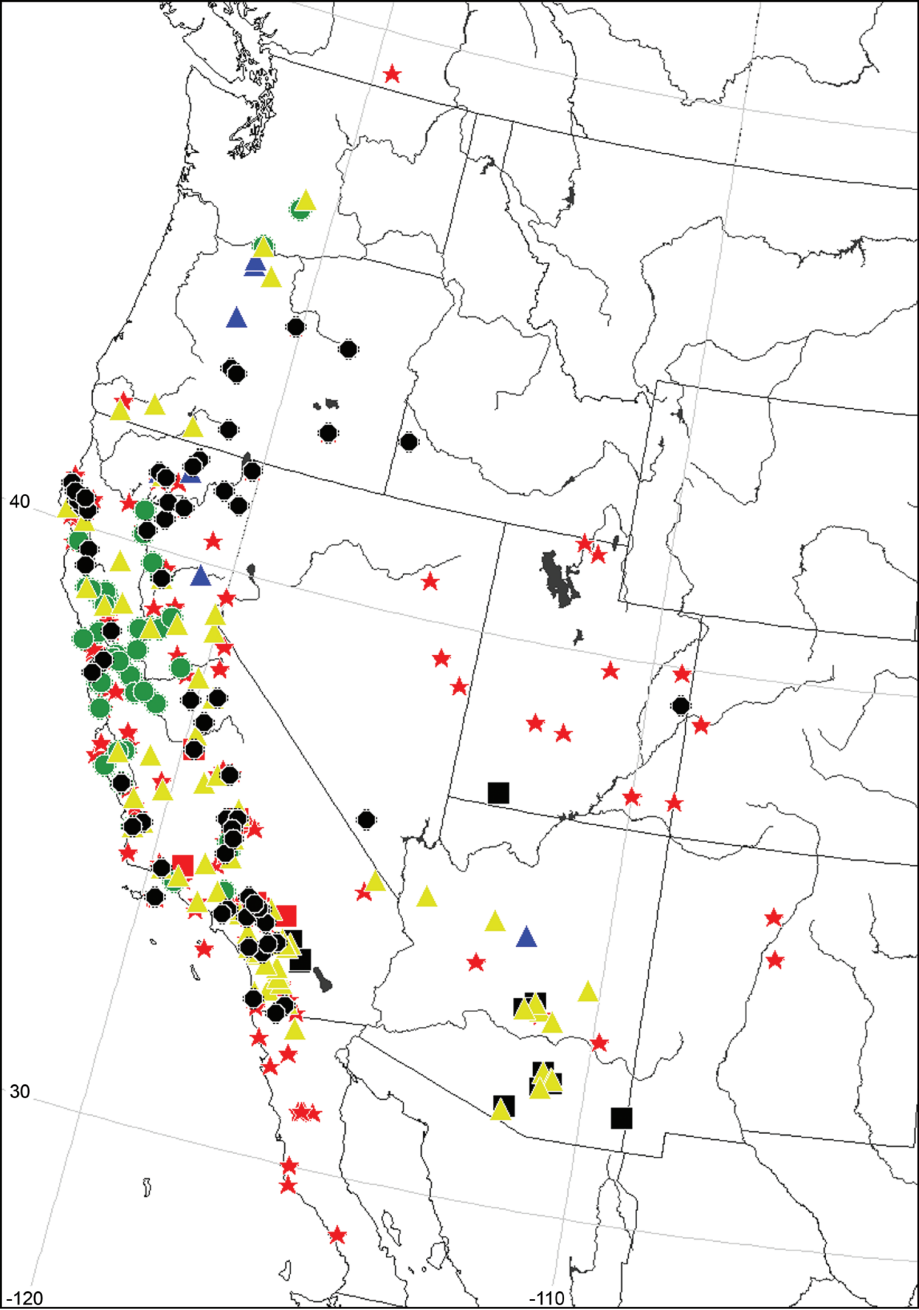
Map of species distributions in western North America created using the Simple Mapper.

Even if your database application does not have integrated mapping tools, the simple ability to export lat-long data will permit the easy visualization of those data and the creation of maps (fig. 3). Some of the tools freely available are the Simple Mapper (Shorthouse 2010), Google Earth, and the Global Mapper of Discover Life (2012). All allow for lat-long data in decimal format to be pasted into the application for production of maps useful for publication or for the preparation of presentations.

### Measurements, images, etc.: Integrating other data sources

As is the case with georeferencing early in the study of specimens, the use of USIs as labels for images, measurement data, and DNA sequences allows these data sources to become an integral part of the data record for the specimens under study, and for tracking those data in an unequivocal manner.

## Data outputs: Organizing data through the power of report writing

### Reports of specimens examined

**Figure 4.**
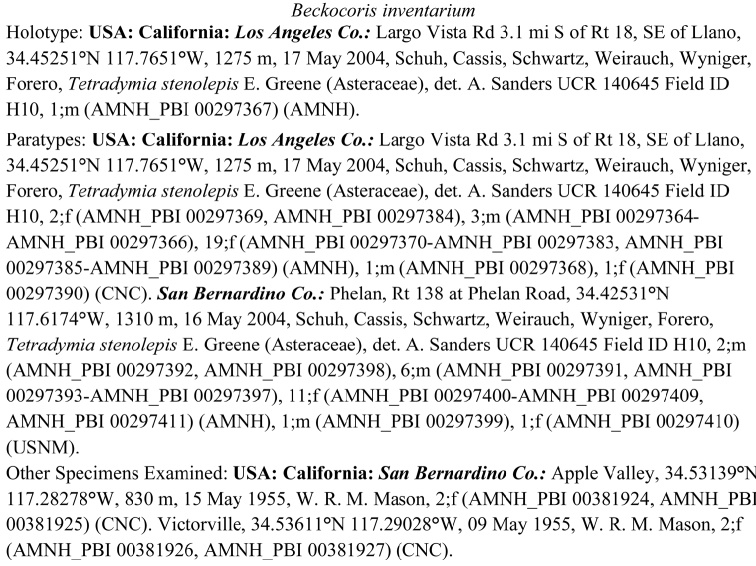
Report of specimens examined, including unique specimen identifiers.

Once specimen data have been captured, checked for accuracy, and georeferenced, the real power of the database for revisionary studies comes from the ability to generate reports. Possibly most valuable is the preparation of reports of specimens examined, a core component of traditional revisions (Fig. 4). The reports can be written, revised, and rewritten in a matter of seconds or minutes, and preclude retyping and reformatting of data; the same can be said for the preparation of maps. Other types of reports, such as species by locality, hosts by species, and range of collection dates–among many other possibilities–are also easily produced and complement the contents of many revisions.

The power of database query languages facilitates the preparation of counts of total specimens examined, specimens examined by museum, specimens dissected, and other summary information that helps to clarify the sources and uses of data.

### Species pages: Integrating all data sources in electronic form

Species pages have become the Internet equivalent of species treatments in traditional print publications. The Encyclopedia of Life (EOL 2012) is centered around this approach and promotes the goal of creating a page for every known species. “Web aggregators” such as Discover Life (2012) produce species pages through highly automated means, providing images, keys, and maps for a very large number of taxa. The research efforts of my colleagues and myself resulted in the creation of the Heteroptera Species Pages (2012; http://research.amnh.org/pbi/heteropteraspeciespage/ ) which assembles available data from a specimen database and creates pages on the Web in real time.

## Descriptive databases: Adding the descriptive component

More has probably been written on the use of descriptive databases in revisionary systematics than has been the case for specimen databases. These products allow for the creation of character descriptions, natural language descriptions, interactive keys, and phylogenetic matrices. The most longstanding version of such a database is DELTA (Dallwitz 2010); a more recent entrant is Lucid Builder (Lucidcentral.org 2012), which has the advantage of employing the TDWG SDD (Structure of Descriptive Data) protocol which allows for the interchange of data with other platforms. One example of moving the descriptive database concept to the Internet is that of Norman Platnick and his NSF-funded team working on the spider family Oonopidae (http://research.amnh.org/oonopidae/index.php ). Descriptive databases and specimen databases are a logical complement to one another. The former require a controlled set of character descriptions in order to function effectively, a time-consuming activity, but one that can pay off handsomely in groups with many species to be described and where ongoing identification of specimens—such as in groups of insects of great economic importance—is a major issue. The latter require the capture of specimen label data, but allow for extensive and continued reuse of those data once acquired.

In my own work, I have created matrices in the program Winclada (Nixon 1999) and used the facilities of the program to output descriptions that can be utilized in publication with minimal editing (e.g., Schuh and Pedraza 2010). As is the case with descriptive databases such as DELTA and Lucid Builder, or with programs such as mx (http://mx.phenomix.org/index.php/Main_Page ), the matrix that is used to prepare descriptions and keys will often not be identical to a matrix well suited to phylogenetic analysis. Nonetheless, the gap between these two uses is oftentimes small, and minimal modification will allow for both matrices to be derived from essentially a single effort.

## Conclusions

In summary, the affordable technology for capture, manipulation, and sharing of specimen data awaits revisers to avail themselves of the opportunity to harness the power of these tools (see Johnson 2007). Experience suggests that seamless integration of revisionary research and database technology will not necessarily take place overnight, but once the logic of using a database as part of revisionary studies is in place, the database will take on the status of a research tool, not just as a way to capture structured specimen data. The time spent on specimen data capture will be quickly repaid through the ability to use those standardized data at every step of the revisionary process, beginning with the standardization of labels by creating the database record of all relevant data at the time of field work, continuing with the creation of maps and reports during the process, and concluding with use of the identical data in the published product. These benefits accrue not only to the individual investigator, but more particularly to research teams where multiple investigators are involved in the preparation of revisions and other specimen-based research products.
